# Supplementary material for: An Insight into Nano Silver Fluoride-Coated Silk Fibroin Bioinspired Membrane Properties for Guided Tissue Regeneration
Source: Polymers (Basel). 2021 Aug 10;13(16):2659. doi: 10.3390/polym13162659 (PMC8401509; doi:10.3390/polym13162659)
Supplement: Supplementary file 1 [file polymers-13-02659-s001.zip › polymers-1323257-supplementary.pdf]

An Insight into Nano Silver Fluoride Coated Silk Fibroin Bioinspired Membrane Properties for Guided Tissue Regeneration

Aditi Pandey[1, §], Tzu-Sen Yang[2, §], Belem Wendimi Fatimata[5], Ta-I Yang[3], Nai-Chia Teng[4], I-Wen Chen[4], Aivaras Kareiva[6], Jen-Chang Yang[1,5,7,8,*]

[1] *Graduate Institute of Nanomedicine and Medical Engineering, College of Biomedical Engineering, Taipei Medical University, Taipei 11052, Taiwan, ROC.*

[2] *Graduate Institute of Biomedical Optomechatronics, Taipei Medical University, Taipei 11031, Taiwan, ROC.*

[3] *Department of Chemical Engineering, Chung-Yuan Christian University, Taoyuan 32023, Taiwan*

[4] *School of Dentistry, College of Oral Medicine, Taipei Medical University, Taipei 11052, Taiwan, ROC.*

[5] *International Ph.D. Program in Biomedical Engineering, College of Biomedical Engineering, Taipei Medical University, Taipei 110-31, Taiwan, ROC.*

[6] *Institute of Chemistry, Vilnius University, Naugarduko 24, LT-03225 Vilnius, Lithuania*

[7] *Research Center of Biomedical Device, Taipei Medical University, Taipei, 110-52, Taiwan, ROC.*

[8] *Research Center of Digital Oral Science and Technology, Taipei Medical University, Taipei, 110-52, Taiwan, ROC.*

[§] Aditi Pandey and Tzu-Sen Yang contributed equally to this work.

***** Correspondence: Dr. Jen-Chang Yang will be responsible for all correspondence and correction of the proofs:

Graduate Institute of Nanomedicine and Medical Engineering, College of Biomedical Engineering, Taipei Medical University, Taipei, Taiwan

250 Wu-Hsing Street, Taipei 110-52, Taiwan, ROC.

Tel: 886-2-2736-1661 ext. 5124; Fax: 886-2-27362295

E-mail: yang820065@tmu.edu.tw

Supplementary Data

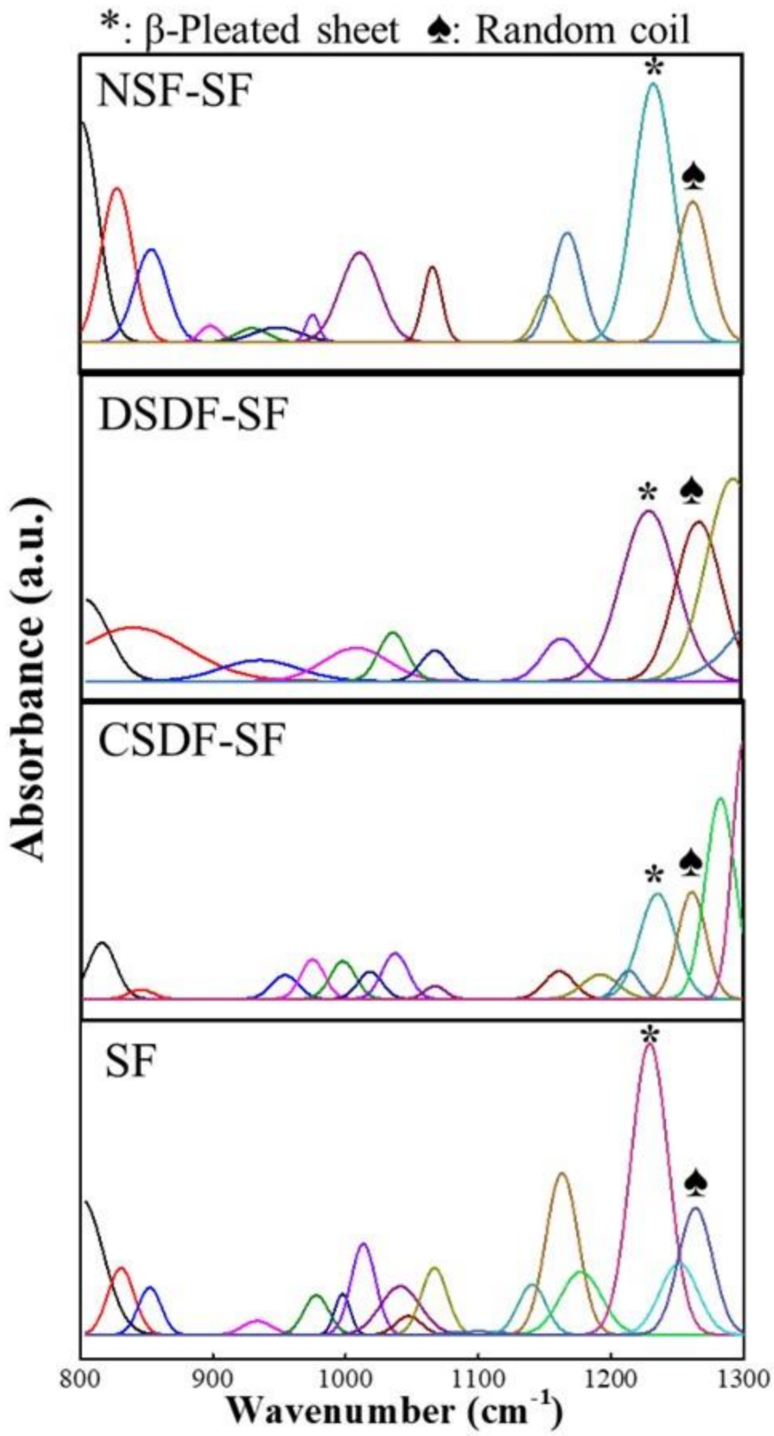

Figure S1: FTIR-ATR spectra of pure SF-based samples, without biomineralization.

Table S1: The β-sheet/random coil ratio of pure non-biomineralized samples

	$\frac{A_{1265}}{A_{1235} + A_{1256}}$	FWHM A_{1235}	FWHM A_{1265}
SF-Pure	31	34.05	22.49
NSF-Pure	33	35.15	29.72
DSDF-Pure	49	49.03	41.26
CSDF-Pure	56	34.44	29.46
